# Jasmonate and Melatonin Act Synergistically to Potentiate Cold Tolerance in Tomato Plants

**DOI:** 10.3389/fpls.2021.763284

**Published:** 2022-01-07

**Authors:** Fei Ding, Liming Ren, Fang Xie, Meiling Wang, Shuoxin Zhang

**Affiliations:** ^1^School of Life Sciences, Liaocheng University, Liaocheng, China; ^2^College of Forestry, Northwest A&F University, Xianyang, China

**Keywords:** jasmonic acid, melatonin, crosstalk, cold tolerance, tomato

## Abstract

Both jasmonic acid (JA) and melatonin (MT) have been demonstrated to play positive roles in cold tolerance, however, whether and how they crosstalk in the cold responses in plants remain elusive. Here, we report that JA and MT act synergistically in the cold tolerance in tomato plants (*Solanum lycopersicum*). It was found that JA and MT were both substantially accumulated in response to cold stress and foliar applications of methyl jasmonate (MeJA) and MT promoted cold tolerance as evidenced by increased Fv/Fm, decreased relative electrolyte leakage (EL) and declined H_2_O_2_ accumulation in tomato plants. Inhibition of MT biosynthesis attenuated MeJA-induced cold tolerance, while inhibition of JA biosynthesis reduced MT accumulation in tomato plants under cold conditions. Furthermore, qRT-PCR analysis showed that the expressions of two MT biosynthetic genes, *SlSNAT* and *SlAMST*, were strongly induced by MeJA, whereas suppression of *SlMYC2*, a master JA signaling regulator, abated the expressions of *SlSNAT* and *SlAMST* under cold stress. Additionally, suppression of *SlMYC2* reduced MT accumulation, decreased Fv/Fm and increased EL in cold-stressed tomato plants. Interestingly, exogenous MT promoted JA accumulation, while inhibition of MT biosynthesis significantly reduced JA accumulation in tomato plants under the cold condition. Taken together, these results suggest that JA and MT act cooperatively in cold tolerance and form a positive feedback loop, amplifying the cold responses of tomato plants. Our findings might be translated into the development of cold-resistant tomato cultivars by genetically manipulating JA and MT pathways.

## Introduction

Unlike animals, plants are sessile and are unable to escape unfavorable growth conditions. Thus, they have to cope with diverse environmental challenges through their life cycles, such as pathogens, extreme temperatures, salinity and drought. Cold, consisting of chilling (0–15°C) and freezing (<0°C), is a recognized environmental stress factor that impairs plant growth and development, restricts geographical distribution of plants in nature, and threatens agricultural productivity of many crop species, especially those of tropical or subtropical origin, including tomato (*Solanum lycopersicum*), maize (*Zea mays*), and soybean (*Glycine max*), among others (Lee et al., 1986; Chinnusamy et al., 2007; Ding et al., 2019). There are several adverse effects of cold stress on plant cells, including (1) cold stress leads to overproduction of reactive oxygen species (ROS), which subsequently causes oxidative damages to nucleic acids, proteins and membranes, and finally disrupts cell functions (Apel and Hirt, 2004; Ruelland et al., 2009); (2) cold stress changes membrane rigidification, which has been proved a key event that induces cold responses (Örvar et al., 2000); (3) cold stress disturbs stability of proteins and inactivates key enzymes involved in essential biological processes. For instance, cold stress impairs photosynthesis by reducing the activity of a Calvin-Benson cycle enzyme sedoheptulose-1,7-bisphosphatase (Ding et al., 2017c). To survive under the cold condition, plants have evolved elaborate mechanisms that improve cold tolerance. One notable example of these mechanisms is the enhancement of antioxidant capacity, which is crucial for ROS homeostasis and mitigation of cold-induced oxidative damages to plant cells (Wang M. et al., 2020). Plants also accumulate more low-molecular-mass solutes under cold stress, including soluble sugars, proline and polyamines, to protect themselves from cold damages (Ruelland et al., 2009; Ding et al., 2017a).

Jasmonates (JAs) are a class of lipid-derived phytohormones, including jasmonic acid and its derivatives, such as methyl jasmonate (MeJA), jasmonoyl-isoleucine (JA-Ile), and 12-OH-JA (Gidda et al., 2003; Świa̧tek et al., 2004; Suza and Staswick, 2008). A growing number of studies show that JAs play crucial roles in plant responses to cold stress (Sharma and Laxmi, 2016; Ding et al., 2020). Upon cold treatment, JA accumulation is increased in *Arabidopsis*, leading to the degradation of JA signaling repressors, JASMONATE-ZIM-DOMAIN PROTEIN 1 (JAZ1) and JAZ4 proteins, which interact with and repress the ICE1-CBF module. Thus, JA improves cold tolerance by activating ICE1-CBF cascade in *Arabidopsis* (Hu et al., 2013). JA also positively regulates cold tolerance in rice, as *HAN1*, which encodes an oxidase catalyzing active JA-Ile to inactive 12-OH-JA-Ile, reduces chilling tolerance (Mao et al., 2019). Emerging evidence indicates that JA also confers cold tolerance in multiple horticultural plant species. In apple (*Malus hupehensis*), JA signaling promotes cold tolerance through the JAZ-BBX37-ICE1-CBF pathway, in which MdJAZ1 and MdJAZ2 interact with BBX37 to repress MdICE1 and two MdCBFs (An et al., 2020). MYC2, a positive regulator of JA signaling, confers cold tolerance by interacting with ICE1 in banana (*Musa acuminata*) (Zhao et al., 2013). JA has also been shown to increase cold tolerance by promoting biosynthesis of osmolytes. In trifoliate orange (*Poncirus trifoliata*), MYC2 activates the transcriptional expression of *PtrBADH-l* to promote glycine betaine biosynthesis, thus conferring increased cold tolerance (Ming et al., 2020). More recently, JA is found to increase chilling tolerance of tomato plants and fruits through MYC2-mediated polyamine biosynthesis (Ding et al., 2021; Min et al., 2021). Though great progress has been made in the understanding of JA-induced cold tolerance, yet the underlying mechanisms by which JA regulates cold tolerance are still not fully understood.

Melatonin (*N*-acetyl-5-methoxytrytamine, MT), structurally similar to indole-3-acetic acid (IAA), is a multifunctional molecule in animals, humans, plants, and algae (Vivien-Roels and Pévet, 1993; Fuhrberg et al., 1996; Ding et al., 2018a,b; Arnao and Hernández-Ruiz, 2019b). The presence of MT in plants was confirmed in 1995 (Dubbels et al., 1995; Hattori et al., 1995) and afterward, plenty of studies have revealed the versatile roles of MT in plant growth, development and stress responses. Due to its multifunctionality and recent identification of a phytomelatonin receptor PMTR1, MT has been proposed as a master regulator and a potential new hormone in plants (Wei et al., 2018; Arnao and Hernández-Ruiz, 2019a). MT has also been shown to crosstalk with different phytohormones to act in a variety of biological processes. For instance, MT interacts with auxin (Weeda et al., 2014; Wang Q. et al., 2016; Wen et al., 2016), ABA (Li et al., 2012; Zhang et al., 2014; Fu et al., 2017; Jahan et al., 2021), gibberellins (Zhang et al., 2014; Jahan et al., 2021; Lv et al., 2021), ethylene (Sun et al., 2015, 2016; Chen et al., 2021), SA (Chen et al., 2021), and brassinosteroids (Hwang and Back, 2018) either by regulating their biosynthesis or signaling pathways. Recently, several lines of evidence suggest that MT is also related to phytohormone jasmonic acid. In watermelon plants (*Citrullus lanatus*), melatonin action on cold tolerance is partly ascribed to MT-induced accumulation of MeJA (Li et al., 2021). In another study, MT is shown to mediate defense response against Huanglongbing, a devastating citrus (*Citrus sinensis*) disease, via crosstalk with JA signaling pathway (Nehela and Killiny, 2020). Moreover, MT promotes root development of copper-stressed melon plants by inhibiting JA biosynthesis (Hu et al., 2020). In addition, MT suppresses JA-induced tomato leaf senescence (Wang et al., 2019). Finally, MT is involved in the MeJA-mediated delay of cassava deterioration during postharvest storage (Liu et al., 2019).

Melatonin has been found to improve cold tolerance in a number of plant species, such as tomato (Ding et al., 2017a,b; Zhou et al., 2019; Wang M. et al., 2020), tea plants (*Camellia sinensis*) (Li et al., 2019), watermelon (Li et al., 2021), bermuda grass (*Cynodon dactylon*) (Shi et al., 2015) and Arabidopsis (Shi and Chan, 2014), among others. As both MT and JA contribute to cold tolerance in plants, we hypothesized that there may exist crosstalk between MT and JA in plant responses to cold stress. In this study, we provide evidence that JA and MT act synergistically to potentiate cold tolerance in tomato plants and we propose that JA and MT form a positive loop that amplifies tomato responses to cold stress.

## Materials and Methods

### Plant Materials

Tomato plants (*Solanum lycopersicum* “MicroTom”), including wild-type tomato plants and *MYC2-RNAi* transgenic tomato plants (Ding et al., 2021) were used in this study. Tomato seeds were germinated and grown in plastic pots filled with peat and vermiculite. Tomato plants were grown in a growth chamber with the following settings: day/night temperature 25°C/20°C, 14-h photoperiod (200 μmol m^–2^ s^–1^ photosynthetic photon flux density), relative humidity ∼60%.

### Treatments

For determination of JA and MT accumulations under cold conditions, at 4-leaf stage, tomato plants were subjected to cold stress at 4°C for 24 h. For determination of cold tolerance affected by exogenous MeJA and MT, tomato plants were pretreated with mock (1/10,000 ethanol), 100 μM MeJA, or 100 μM MT 12 h prior to cold treatment. Tomato plants were treated for 24 h for physiological assessment and 60 h for phenotype analysis. To block the biosynthesis of JA, a JA biosynthesis inhibitor DIECA (diethyldithiocarbamic acid) was used. Tomato plants were treated with 2 mM DIECA 12 h before being subjected to cold stress. To block the biosynthesis of MT, 100 μM CPA (*p*-chlorophenylalanine, a MT biosynthesis inhibitor) was used (Li et al., 2021) to treat tomato plants prior to cold stress.

To assess the induction of MT biosynthetic genes by MeJA, fully expanded young leaves were detached from tomato plants at 4-leaf stage and were incubated in 50 μM MeJA. Leaves were harvested at 0, 2, 4, 8, and 12 h following MeJA treatment and were used for qRT-PCR analysis. To determine the regulation of MT biosynthesis by SlMYC2, detached leaves from two *SlMYC2-RNAi* transgenic lines were incubated in 50 μM MeJA for 12 h and were collected for qRT-PCR analysis. Similarly, to assess the induction of JA biosynthetic genes by MT, detached leaves were incubated in 50 μM MT and leaves were collected at 0, 2, 4, 8, 12, and 24 h following MeJA treatment.

### Cold Tolerance Assays

Cold tolerance was evaluated by measuring Fv/Fm, EL, and H_2_O_2_ accumulation. Fv/Fm was obtained with a portable chlorophyll fluorometer (PAM-2000, Walz, Germany) as described in a previous study (Ding et al., 2016). Tomato plants were first dark adapted for 30 min and the minimal fluorescence (Fo) was obtained. Then a saturating pulse was used to yield the maximal fluorescence (Fm). Finally, the maximum quantum efficiency (Fv/Fm) was calculated.

Electrolyte leakage measurement was performed following a previous study (Ding et al., 2018c). Leaf samples were collected and incubated in deionized water and the conductivity of the incubated solution was measured as C1. Leaf samples were then boiled and the conductivity of the solution was measured as C2. The relative EL was calculated as the ratio of C1/C2.

Quantification of H_2_O_2_ was performed according to previous studies (Patterson et al., 1984; Wang M. et al., 2020). Leaf samples were ground with 5% (w/v) trichloroacetic acid. The resulting homogenate was centrifuged and the supernatant was mixed with TiCl_2_ to form the Ti–H_2_O_2_ complex, which was further precipitated using ammonia solution. The resulting precipitate was resuspended in H_2_SO_4_ and the absorbance of the solution was measured at 410 nm and was used for the calculation of H_2_O_2_ content.

### Quantification of Melatonin

Quantification of MT was performed by High-Performance Liquid Chromatography (HPLC) as previously described (Ding et al., 2017a). Briefly, frozen leaf samples were homogenized in chloroform and melatonin was extracted at 4°C in the dark. The crude extraction was then centrifuged at 4,000 × *g* for 5 min and the chloroform phase was purified using a C18 solid-phase extraction (SPE) cartridge. The extract was evaporated under N_2_ gas and the pellet was resuspended in methanol for HPLC analysis. The HPLC system was equipped with a 5 μm Hypersil ODS column (C18) and a fluorescence detector. The mobile phase was methanol and was delivered at a flow rate of 1.0 mL min^–1^. Twenty μL samples were injected into the system. For melatonin detection, the excitation wavelength was set at 280 nm and the emission wavelength was set as 348 nm.

### Quantification of Jasmonic Acid

Quantification of JA was performed following published procedures with slight modifications (Wang F. et al., 2016). Briefly, leaf samples were ground into fine powder in liquid nitrogen and homogenized in ethyl acetate. The homogenate was shaken at 4°C overnight. Then, the homogenate was centrifuged at 18,000 × *g* for 10 min. The supernatant was collected and the pellet was resuspended with ethyl acetate, followed by centrifugation for 10 min at 18,000 × *g* and the supernatant was collected. The supernatants were mixed and evaporated to dryness using nitrogen gas. The residue was resuspended in methanol and centrifuged at 18,000 × *g* for 2 min, and the supernatants were subjected to analysis by HPLC. HPLC analysis was conducted with a 3.5 μm Agilent ZORBAX XDB column (C18). The mobile phase was a mixture of 0.1% formic acid and methanol at a flow rate of 0.3 mL min^–1^. The column temperature was set at 40°C and 20 μL sample was injected into the system.

### Quantification of Transcript Abundance by Quantitative Real-Time PCR

Quantification of transcript abundance was performed by quantitative real-time PCR (qRT-PCR). Briefly, total RNA was extracted from detached leaves treated with MeJA using RNAprep Pure Plant Kit (QIAGEN) according to manufacturer’s instructions. The RNA was then used for cDNA synthesis. qRT-PCR was performed using a Premix Ex Taq kit (TaKaRa, Dalian, China). Tomato *ACTIN2* was used as an internal control. Specific primers used in this study were listed in Supplementary Table 1.

### Statistical Analysis

The experiments were performed with three independent replicates. The values were presented as means ± standard deviations (SDs). The data were analyzed by means of ANOVA, and *p*-values < 0.05 were considered significantly different according to Tukey’s test. Different letters represent significant difference at *p* < 0.05 in each figure.

## Results

### Cold Stress Promotes the Accumulation of Jasmonic Acid and Melatonin in Tomato Plants

**FIGURE 1:**
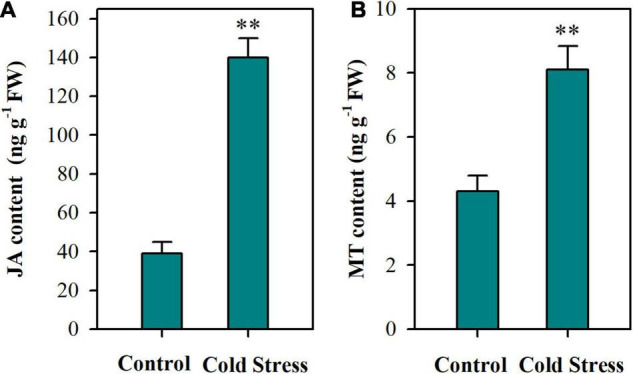
Contents of JA and MT in tomato leaves under cold stress. **(A)** JA content; **(B)** MT content. At 4-leaf stage, tomato plants were subjected to cold stress at 4°C for 24 h. Following treatment, leaves were harvested for determination of JA and MT content. Data are mean values of three replicates ± SD. Asterisks represent significant difference at *P* < 0.01 according to Student’s *t*-test.

To investigate the actions of JA and MT in the cold tolerance of tomato plants, we first examined the endogenous accumulation of JA and MT in tomato plants following a 24 h cold treatment. It was shown that tomato plants under cold conditions accumulated remarkably more JA than those under control conditions (Figure 1A). Likewise, MT production was significantly enhanced by cold treatment, with MT content being increased by 88% (Figure 1B). These results indicate that both JA and MT are involved in the responses to cold stress in tomato plants.

### Exogenous Jasmonic Acid and Melatonin Enhance Cold Tolerance in Tomato Plants

**FIGURE 2:**
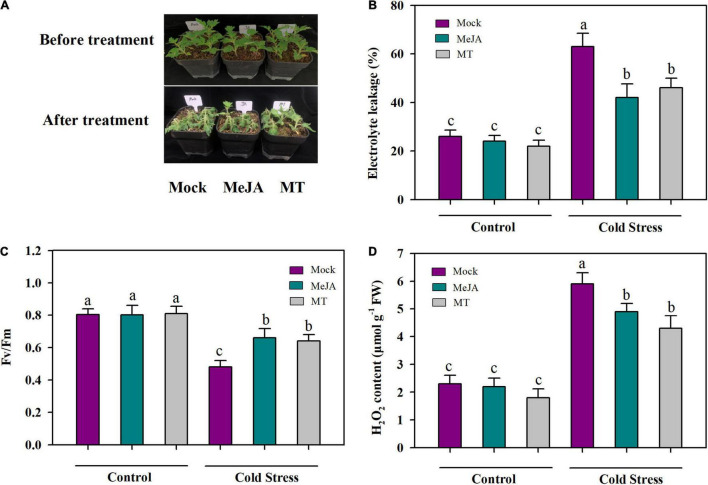
MeJA and MT promote cold tolerance. **(A)** Representative MeJA- and MT-treated tomato plants after cold stress. **(B)** Fv/Fm; **(C)** EL; **(D)** H_2_O_2_ content. At 4-leaf stage, tomato plants were treated with mock, 100 μM MeJA or 100 μM MT 12 h before they were subjected to cold stress at 4°C. Tomato plants were treated for 24 h for physiological assessment and 60 h for phenotype analysis. Data are mean values of three replicates ± SD. Data for Fv/Fm are mean values from 12 leaves. Different letters represent significant difference at *P* < 0.05 according to Tukey’s test.

Having found that accumulations of JA and MT were increased in response to cold stress, we next assessed the roles of JA and MT in cold tolerance in tomato plants by applying exogenous MeJA and MT to tomato leaves and measuring maximum photochemical efficiency (Fv/Fm), relative electrolyte leakage (EL) and accumulation of hydrogen peroxide (H_2_O_2_). It was observed that cold stress markedly decreased Fv/Fm in tomato plants, while exogenous MeJA and MT alleviated cold-induced inhibition of Fv/Fm. Under cold stress, compared with foliar application of mock, application of MeJA and MT increased Fv/Fm by 41.7 and 33.3%, respectively (Figure 2B). Membrane integrity is closely associated with cold tolerance in plants, so we next examined the relative electrolyte leakage (EL) to evaluate membrane integrity of tomato plants subjected to different treatments. The results showed that cold stress increased EL by 63% in tomato leaves; However, application of MeJA decreased EL by 33% and application of MT decreased EL by 27% in comparison with application of mock under cold stress (Figure 2C), indicating that MeJA and MT exerted protective effective on membranes in tomato leaves under the cold condition. As cold stress generally results in hyperaccumulation of ROS, we also examined the level of H_2_O_2_ in cold-stressed tomato plants. It was found that the content of H_2_O_2_ was enhanced in tomato plants exposed to cold stress compared with that in tomato plants under control growth conditions, whereas application of MeJA and MT significantly reduced the level of H_2_O_2_ in tomato plants under cold stress (Figure 2D). Altogether, these results support the crucial roles of MeJA and MT in the cold tolerance of tomato plants.

### Methyl Jasmonate Increases Cold Tolerance Partly Through Melatonin in Tomato Plants

**FIGURE 3:**
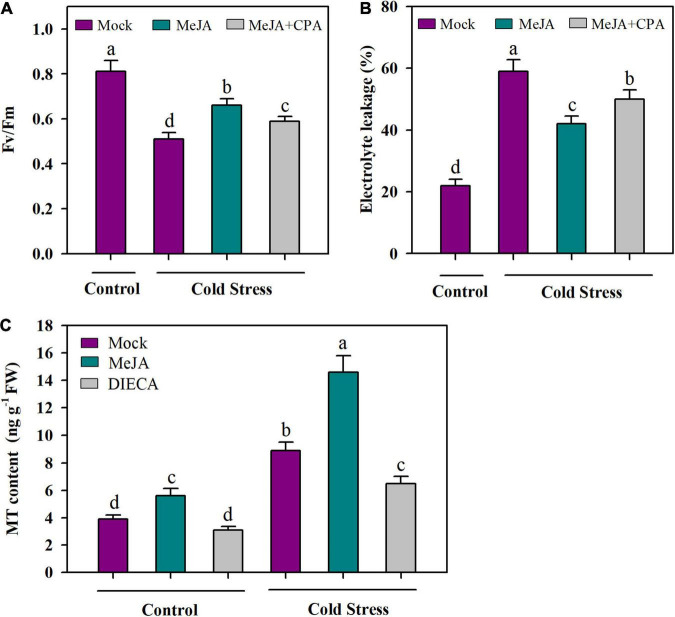
MeJA-induced cold tolerance partly depends on MT. **(A)** Fv/Fm; **(B)** EL; **(C)** MT content. For determination of Fv/Fm and EL, tomato plants at 4-leaf stage were treated with mock, 100 μM MeJA or 100 μM MeJA plus 100 μM CPA (*p*-chlorophenylalanine, a MT biosynthesis inhibitor) 12 h before they were subjected to cold stress at 4°C for 24 h. For determination of MT content, at 4-leaf stage, tomato plants were treated with mock, 100 μM MeJA or 2 mM DIECA (diethyldithiocarbamic acid, a MT biosynthesis inhibitor) 12 h before they were subjected to cold stress at 4°C for 24 h. Data are mean values of three replicates ± SD. Data for Fv/Fm are mean values from 12 leaves. Different letters represent significant difference at *P* < 0.05 according to Tukey’s test.

To understand the role of MT in JA-mediated cold tolerance, we treated tomato plants with MeJA and a MT biosynthesis inhibitor (CPA), and assessed the changes in cold tolerance. Compared with application of mock under cold conditions, foliar application of MeJA significantly increased Fv/Fm by 30%, however, this MeJA-mediated increase in Fv/Fm was attenuated by application of CPA in tomato plants, with the increase being reduced by 14% (Figure 3A). We also performed EL analysis and the results showed that under cold stress, MeJA treatment led to decreased EL, while the combined treatment of MeJA and CPA abated the effect of MeJA on EL (Figure 3B). These results imply that MeJA-induced cold tolerance may be partly ascribed to MT.

As we have found that MeJA may act through MT in the cold tolerance of tomato plants, we next asked whether MeJA plays a role in the accumulation of MT in cold-stressed tomato plants. To answer this question, we treated tomato plants with MeJA and a JA biosynthesis inhibitor (diethyldithiocarbamic acid, DIECA) and determined the content of MT. It was observed that under normal growth conditions at 25°C, application of MeJA significantly increased MT content, while application of DIECA only marginally reduced MT content. Notably, under cold conditions at 4°C, MeJA largely boosted the accumulation of endogenous MT compared with mock, with the increase being 64%, whereas DIECA significantly inhibited MT accumulation of MT (Figure 3C). These results suggest that JA may have a crucial role in the biosynthesis of MT in tomato plants under cold stress.

### Methyl Jasmonate Positively Regulates the Transcriptional Expression of Melatonin Biosynthetic Genes in Tomato Plants

**FIGURE 4:**
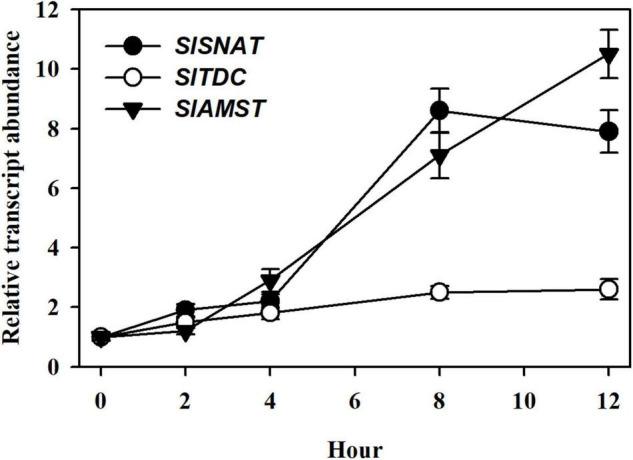
Relative transcript abundance of MT biosynthetic genes, including *SlSNAT*, *SlTDC*, and *SlAMST* in response to MeJA. At 4-leaf stage, fully expanded young leaves were detached from tomato leaves and were incubated in 50 μM MeJA for 12 h. Following MeJA treatment, leaves were collected at 0, 2, 4, 8, and 12 h for relative expression analysis by qRT-PCR. The expression level of leaf samples collected at 0 h was set to 1, and the relative expression levels of the rest of samples were calculated accordingly. Data are mean values of three replicates ± SD.

To explore the potential mechanisms of JA-mediated MT accumulation, we incubated detached tomato leaves in 50 μM MeJA for 12 h and determined the transcript abundance of three tomato genes through qRT-PCR analysis, including *SlSNAT* (Solyc10g074910) (Wang X. et al., 2020), *SlTDC* (Solyc09g064430) (Li et al., 2016) and *SlAMST* (Solyc03g080180) (Xu et al., 2016), which have been demonstrated to act in MT biosynthesis in tomato plants. Following a 4 h MeJA treatment, the relative expression between *SlSNAT*, *SlTDC* and *SlAMST* did not exhibit significant difference. At 8 and 12 h, *SlSNAT* and *SlAMST* were intensively induced, while *SlTDC* remained at a low level of relative expression (Figure 4), suggesting that *SlSNAT* and *SlAMST* are two major MT biosynthetic genes responsive to MeJA. These results imply that the observed MT accumulation by MeJA may rely, in part, on JA-induced expression of *SlSNAT* and *SlAMST*.

### Suppression of *SlMYC2* Represses Methyl Jasmonate-Induced Expression of Melatonin Biosynthetic Genes in Tomato Plants

**FIGURE 5:**
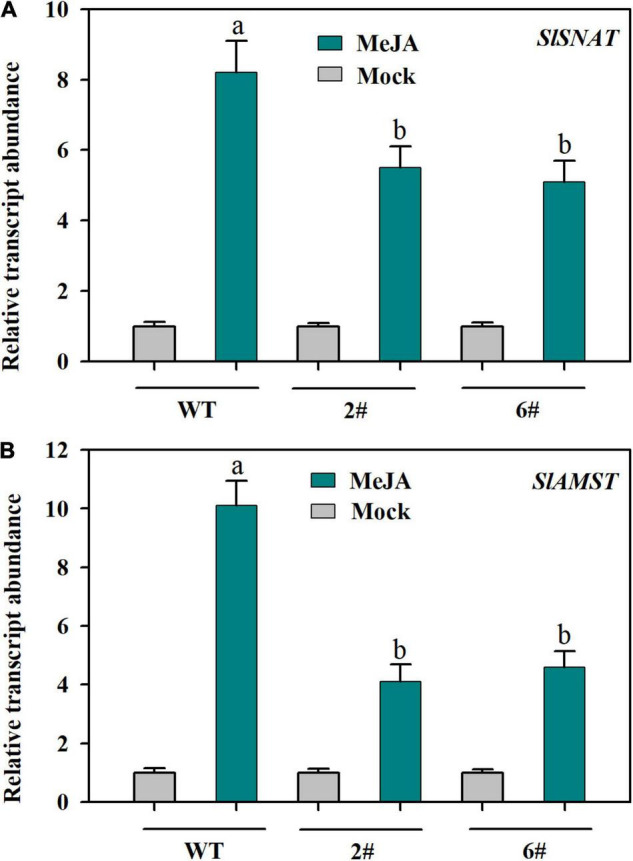
Relative transcript abundance of *SlSNAT* and *SlAMST* as affected by knockdown of *SlMYC2*. **(A)** *SlSNAT*; **(B)** *SlAMST*. At 4-leaf stage, fully expanded young leaves were detached from wild-type and *SlMYC2-RNAi* transgenic tomato plants and were incubated in 50 μM MeJA for 12 h. Following MeJA treatment, leaves were collected for relative expression analysis by qRT-PCR. The expression level of mock-treated samples was set to 1, and the relative expression level of MeJA-treated samples was calculated accordingly. Data are mean values of three replicates ± SD. Different letters represent significant difference at *P* < 0.05 according to Tukey’s test.

To investigate whether MeJA-induced expression of *SlSNAT* and *SlAMST* depends on JA signaling, we utilized two *SlMYC2-RNAi* transgenic lines, in which MYC2, a master transcriptional regulator of JA signaling, was substantially suppressed (Ding et al., 2021). In response to MeJA, the relative expression of *SlSNAT* was dramatically increased in both wild-type plants and two *SlMYC2-RNAi* transgenic lines, however, the expression level was significantly reduced in *SlMYC2-RNAi* plants compared with that in wild-type plants (Figure 5A). Similar results were also observed for *SlAMST* (Figure 5B). These results suggest that *SlSNAT* and *SlAMST* may be subjected to regulation by MYC2-dependent JA signaling.

### Suppression of *SlMYC2* Decreases Melatonin Accumulation and Cold Tolerance in Tomato Plants

**FIGURE 6:**
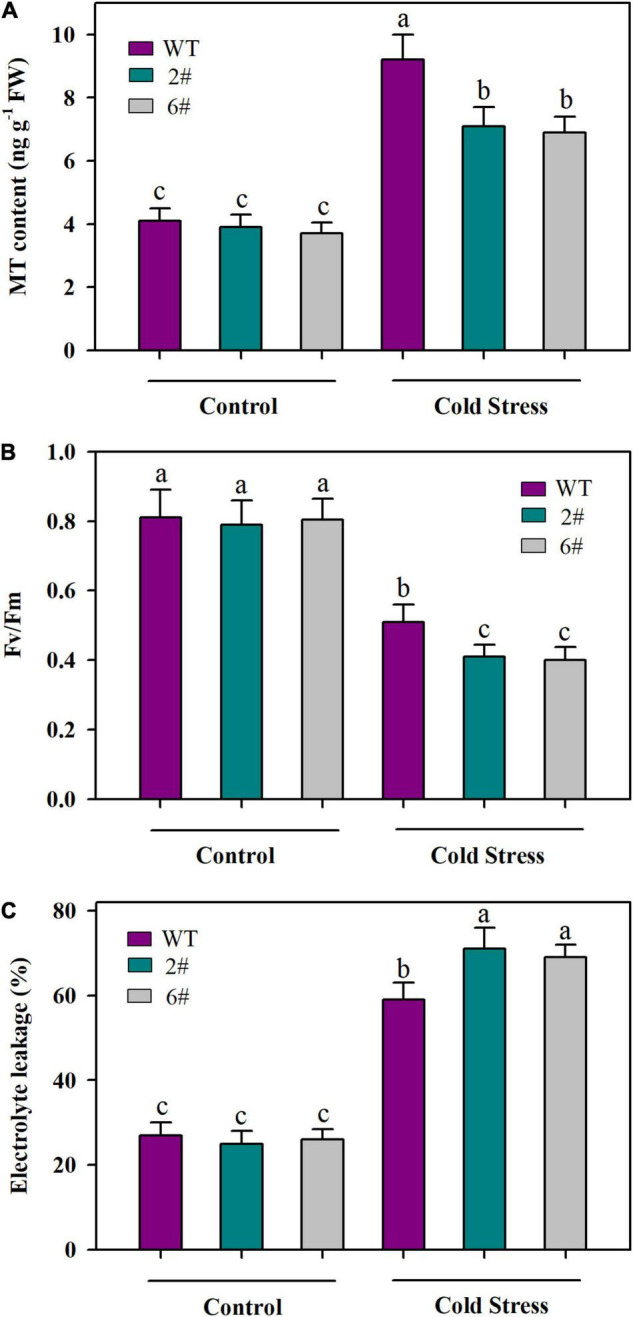
MT content and cold tolerance as affected by knockdown of *SlMYC2*. **(A)** MT content; **(B)** Fv/Fm; **(C)** EL. At 4-leaf stage, wild-type and *SlMYC2-RNAi* transgenic tomato plants were subjected to cold stress at 4°C for 24 h. Following cold treatment, leaves were collected for determination of MT content, Fv/Fm and EL. Data are mean values of three replicates ± SD. Data for Fv/Fm are mean values from 12 leaves. Different letters represent significant difference at *P* < 0.05 according to Tukey’s test.

To further verify that JA signaling is important for MT accumulation and the responses to cold stress in tomato plants, we analyzed the MT content, Fv/Fm and EL in wild-type and *SlMYC2-RNAi* transgenic plants under cold conditions. In accordance with decreased expression of *SlSNAT* and *SlAMST* that we observed in our last experiment, MT content was significantly reduced in *SlMYC2-RNAi* transgenic plants compared with that in wild-type plants under cold stress (Figure 6A). Consistently, *SlMYC2-RNAi* transgenic plants displayed lower Fv/Fm and higher EL than wild-type plants under cold conditions (Figures 6B,C). These results substantiate the idea that JA-mediated cold tolerance may depend on MYC2-regulated MT biosynthesis in tomato plants.

### Melatonin Promotes the Accumulation of Jasmonic Acid in Tomato Plants Under Cold Conditions

**FIGURE 7:**
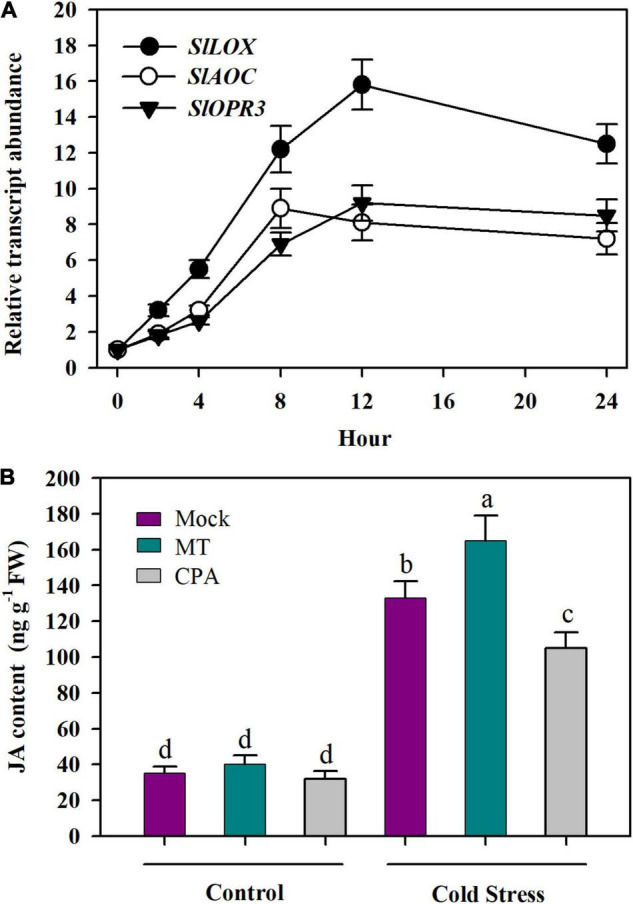
MT promotes the accumulation of JA under cold stress. **(A)** Relative transcript levels of JA biosynthesis genes; **(B)** JA content. For determination of relative transcript levels of JA biosynthesis genes, fully expanded young leaves were detached from tomato leaves at 4-leaf stage and were incubated in 50 μM MT for 12 h. Following MT treatment, leaves were collected at 0, 2, 4, 8, 12, and 24 h for relative expression analysis by qRT-PCR. For determination of JA content, tomato plants at 4-leaf stage were treated with mock solution, 100 μM MT or 100 μM CPA 12 h before they were subjected to cold stress at 4°C for 24 h. Data are mean values of three replicates ± SD. Different letters represent significant difference at *P* < 0.05 according to Tukey’s test.

The finding that JA enhanced MT biosynthesis in tomato plants under cold stress prompted us to ask whether MT promoted JA biosynthesis in tomato plants in a feedback manner. To resolve this question, we first incubated detached tomato leaves in 50 μM MT for 12 h and determined the transcript levels of three JA biosynthetic genes in tomato leaves, including *SlLOX* (Solyc03g122340), *SlAOC* (Solyc02g085730), and *SlOPR3* (Solyc07g007870). It was shown that following MT treatment, the relative transcript levels of three selected JA biosynthetic genes were increased (Figure 7A), suggesting the potential regulation of JA biosynthesis by MT.

To further ascertain that MT regulates the biosynthesis of JA, we treated tomato leaves with MT or its biosynthesis inhibitor CPA and determined the JA content. The results showed that foliar application of MT slightly increased JA accumulation under control growth conditions, while MT significantly enhanced JA accumulation under cold stress, with the increase being 24%. Of particular note is the inhibition of endogenous MT by its biosynthesis inhibitor CPA reduced JA accumulation in tomato plants, with the JA level being reduced by 17% under cold stress (Figure 7B). These results provide evidence that MT plays a positive role in the biosynthesis of JA in tomato leaves under cold conditions.

## Discussion

Cold is an adverse environmental factor that poses threats to plants of tropical or subtropical origin. Cold stress inhibits plant growth and development, and causes severe losses of crop yields. Understanding the responses to cold stress in plants is important for development of cold resistant crop cultivars. Breeding cold-hardy crops is the most effective strategy to mitigate cold stress in agricultural practices. JAs are a group of phytohormones and have been implicated in defense against pathogens and herbivores, as well as abiotic stresses, such as drought, salinity and cold (Kazan, 2015; Wang J. et al., 2020). A great many studies have demonstrated that JA plays a key role in cold tolerance in a diversity of plant species, including tomato (Ding et al., 2021), apple (An et al., 2020), trifoliate orange (Ming et al., 2020), rice (Lee et al., 1996) and Arabidopsis (Hu et al., 2013), among others. Melatonin (MT), a newly identified plant growth regulator, has been shown to play versatile roles in plant growth and development, and resistance to biotic and abiotic stresses. Accumulating evidence suggest that MT acts in the response to cold stress in plants (Wu et al., 2021). Both JA and MT regulate cold responses in plants, however, whether and how they crosstalk in the cold tolerance remains to be determined. In this study, we explored the potential crosstalk between JA and MT in the cold tolerance of tomato, which originated from tropical regions and is cold sensitive. We demonstrated that JA and MT form a positive feedback loop to promote respective biosynthesis and boost cold tolerance in tomato plants.

Previous studies have established that JA and MT are involved in the responses to cold tolerance in plants (Sharma and Laxmi, 2016; Tiwari et al., 2020). Our work provides several lines of evidence that both JA and MT are of importance in the cold tolerance in tomato plants. Firstly, levels of endogenous JA and MT were substantially increased by cold stress. Secondly, exogenous MeJA and MT significantly increased Fv/Fm and decreased EL. Finally, application of MeJA or MT led to reduced production of H_2_O_2_ under the cold condition. Though these results confirm the pivotal role of JA and MT in the cold tolerance, whether and how they crosstalk in tomato cold response is a yet-to-be answered question. In our attempt to address this question, we first found that MeJA enhanced cold tolerance, while CPA attenuated MeJA-induced cold tolerance (Figures 3A,B), suggesting that JA and MT are associated in the cold response, and that MT may act downstream of MeJA mediating MeJA-triggered cold tolerance in tomato plants. Additionally, MeJA increased MT accumulation, whereas a JA biosynthesis inhibitor DIECA decreased MT accumulation under the cold condition (Figure 3C). Together with previous reports that MeJA promotes MT accumulation to delay cassava deterioration and to improve watermelon cold tolerance (Liu et al., 2019; Li et al., 2021), these results support the interactions between JA and MT in tomato plants. These results also imply that JA-induced MT biosynthesis may be an important adaptive strategy in plants subjected to cold stress.

Though we observed that MeJA promoted MT biosynthesis, the relevant mechanisms or the major factors involved remain unclear. In plants, MT biosynthesis involves five major enzymes, catalyzing four main sequential steps. Briefly, tryptophan decarboxylase (TDC) first converts tryptophan to tryptamine, which is then catalyzed by tryptamine-5-hydroxylase (T5H) to produce serotonin. Conversion of serotonin to melatonin involves two independent pathways in plants. In one pathway, serotonin-*N*-acetyltransferase (SNAT) converts serotonin into *N*-acetylserotonin, which is then catalyzed to melatonin by *N*-acetylserotonin methyltransferase (ASMT) or caffeic acid O-methyltransferase (COMT). In the other pathway, ASMT/COMT first catalyzes serotonin into 5-methoxytryptamine, which is further converted to melatonin by SNAT (Wu et al., 2021). *SlSNAT*, *SlTDC* and *SlAMST* have been demonstrated to function in MT biosynthesis in tomato plants (Li et al., 2016; Xu et al., 2016; Wang X. et al., 2020). We found that *SlSNAT* and *SlAMST*, rather than *SlTDC*, were predominantly induced following MeJA treatment, implying that JA-induced expression of *SlSNAT* and *SlAMST* may contribute to cold-induced MT accumulation. It is thus reasonable to conclude that cold induces JA accumulation, as we observed in this study, which subsequently triggers the expression of *SlSNAT* and *SlAMST*, ultimately giving rise to increased melatonin accumulation. In an attempt to further understand how JA induces expression of *SlSNAT* and *SlAMST*, as well as MT biosynthesis, we took advantage of two *MYC2-RNAi* transgenic lines, in which a key JA signaling regulator, *SlMYC2*, was knocked down (Ding et al., 2021). Compared with their wild-type counterparts, *MYC2-RNAi* transgenic plants displayed lower transcript levels of *SlSNAT* and *SlAMST*, in response to MeJA treatment. Of particular note is that under cold conditions *MYC2-RNAi* tomato plants accumulated significantly less MT than wild-type plants. Furthermore, downregulation of *SlMYC2* attenuates JA-induced cold tolerance. Our results thus support that SlMYC2-dependent JA signaling is critical for MT biosynthesis in cold-stressed tomato plants, adding another line of evidence for JA-induced MT accumulation and cold tolerance in tomato plants. However, it is worth noting that further work on the mechanistic explanation of SlMYC2-regulated expression of *SlSNAT* and *SlAMST* is required to fully understand JA-induced accumulation of MT in tomato plants under cold conditions.

As discussed above, it is convincing that under the cold condition, JA is, at least partially, responsible for the observed increase in MT accumulation. JA and MT were both accumulated in response to cold stress. Having found that JA increases MT accumulation, we are not clear whether MT increases JA accumulation under cold conditions at this stage. It has been well studied that JAs are produced from α-linolenic acid through a series of steps catalyzed by LOXs, AOS, AOC, and OPR (Wasternack and Song, 2017). Our results revealed that expression of *SlLOX*, *SlAOC*, and *SlOPR3* was profoundly stimulated in response to MT, suggesting the involvement of MT in JA biosynthesis. Furthermore, MT increased JA accumulation, while CPA reduced its accumulation. Though it remains unclear how MT stimulated the expression of JA biosynthesis genes and subsequent increase in JA accumulation, these results verify that MT positively regulates the biosynthesis of JA in tomato plants under the cold condition. Regulation of JA biosynthesis occurs at multilayers, including at the transcriptional, post-transcriptional and post-translational levels. Previous studies have demonstrated that there exists a positive feedback loop in expression of JA biosynthesis genes by JA. In addition, MYC2, the master regulator of JA signaling, is able to directly bind to the promoter of JA biosynthetic genes (Wasternack and Song, 2017; Zhang et al., 2020), indicating the complexity of JA biosynthesis regulation. In our work, we did not examine the impact of MT on SlMYC2 expression, making it impossible to determine whether MT-induced JA accumulation depends on SlMYC2. Therefore, future studies should be directed to identify components that are regulated by MT and contribute to JA biosynthesis.

## Conclusion

**FIGURE 8:**
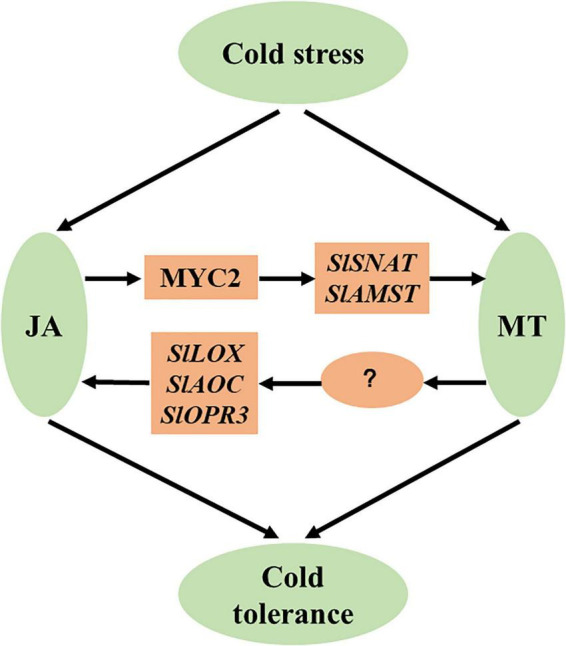
A simplified model depicting the synergistic action of JA and MT in the cold responses of tomato plants. Cold stress increases JA accumulation, leading to elevated MT biosynthesis via MYC2-activated *SlSNAT* and *SlAMST*. Consequently, increased MT accumulation potentiates cold tolerance in tomato plants. JA-induced MT accumulation promotes JA biosynthesis, resulting in a positive feedback loop between JA and MT biosynthesis, which amplifies cold responses in tomato plants.

We demonstrate in the present study that JA and MT act synergistically in the cold tolerance of tomato plants. We propose a working model to describe the crosstalk between JA and MT in the enhancement of cold tolerance (Figure 8). Cold stress increases JA accumulation, which leads to elevated MT biosynthesis via MYC2-activated *SlSNAT* and *SlAMST*. Consequently, increased MT accumulation potentiates cold tolerance in tomato plants. Notably, JA-induced MT accumulation promotes JA biosynthesis, resulting in a positive feedback loop between JA and MT biosynthesis, which amplifies cold responses in tomato plants. Our work thus provides insight into the mechanisms of cold-induced accumulation of JA and MT and their crosstalk in the cold responses in tomato plants. Our findings could be translated into the development of cold-resistant tomato cultivars by genetically manipulating JA and MT pathways.

## Data Availability Statement

The original contributions presented in the study are included in the article/Supplementary Material, further inquiries can be directed to the corresponding author/s.

## Author Contributions

MW, FD, and SZ designed the study and performed the data analysis. FD, LR, FX, and MW conducted the experiments. MW and FD drafted the manuscript. MW and SZ edited the manuscript. All authors approved the publication of current version of the manuscript.

## Conflict of Interest

The authors declare that the research was conducted in the absence of any commercial or financial relationships that could be construed as a potential conflict of interest.

## Publisher’s Note

All claims expressed in this article are solely those of the authors and do not necessarily represent those of their affiliated organizations, or those of the publisher, the editors and the reviewers. Any product that may be evaluated in this article, or claim that may be made by its manufacturer, is not guaranteed or endorsed by the publisher.
