# Supplementary material for: Jasmonate and Melatonin Act Synergistically to Potentiate Cold Tolerance in Tomato Plants
Source: Front Plant Sci. 2022 Jan 7;12:763284. doi: 10.3389/fpls.2021.763284 (PMC8776829; doi:10.3389/fpls.2021.763284)
Supplement: Supplementary file 1 [file Table_1.DOCX]

Supplementary Table S1 Primers used for the determination of transcript abundance.

| ID | Primer sequence (5’-3’) |
| --- | --- |
| *SlSNAT-*F | GGAGGAGATGTTGATGTGTATG |
| *SlSNAT*-R | TGCATGATCTGATGTTGCAC |
| *SlAMST-*F | TCAACAAGGCATATGGAATGAC |
| *SlAMST-*R | CAACAATGGAGTTGAGTCCTTC |
| *SlTDC-*F | GATCACTGAAGCTGTGGATGG |
| *SlTDC-*R | CCAATGAGAACTTCCGAGG |
| *SlACTIN2*-F | TTGCTGACCGTATGAGCAAG |
| *SlACTIN2*-R | GGACAATGGATGGACCAGAC |
